# Case report: The outcomes of neoadjuvant immunotherapy combined with chemotherapy in pulmonary sarcomatoid carcinoma: case series and literature review

**DOI:** 10.3389/fimmu.2024.1467755

**Published:** 2024-11-26

**Authors:** Xiaokang Guo, Jingjing Wang, Daosheng Li, Bin Wang, Hui Zhu, Hongbo Guo

**Affiliations:** ^1^ Department of Thoracic Surgery, Shandong Cancer Hospital and Institute, Shandong First Medical University and Shandong Academy of Medical Sciences, Jinan, Shandong, China; ^2^ Department of Pathology, Shandong Cancer Hospital and Institute, Shandong First Medical University and Shandong Academy of Medical Sciences, Jinan, Shandong, China; ^3^ Department of Pathology, the Afliated Taian City Central Hospital of Qingdao University, Taian, Shandong, China; ^4^ Department of Thoracic Surgery, Changyi People’s Hospital, Weifang, Shandong, China; ^5^ Department of Radiation Oncology, Shandong Cancer Hospital and Institute, Shandong First Medical University and Shandong Academy of Medical Sciences, Jinan, Shandong, China

**Keywords:** case report, pulmonary sarcomatoid carcinoma, immunotherapy, targeted therapy, biomarkers

## Abstract

**Background:**

Pulmonary sarcomatoid carcinoma (PSC) is a highly aggressive malignancy with a significant risk of recurrence even after surgical intervention, leading to a dismal prognosis. In recent years, perioperative immunotherapy has demonstrated promising results in resectable non-small cell lung cancer (NSCLC). However, there is a lack of studies reporting the efficacy of perioperative immunotherapy in PSC.

**Case presentation:**

We report the clinical outcomes of four patients diagnosed with locally advanced PSC who underwent neoadjuvant immunotherapy in combination with chemotherapy from 2021 to 2023 in our hospital. Prior to surgery, these patients received 2 to 4 cycles of neoadjuvant treatment. Post-treatment imaging assessments indicated a partial response (PR) in all cases, and each patient successfully achieved R0 resection. Pathological evaluations demonstrated significant pathological responses: one patient attained Pathological Complete Response (PCR), two patients exhibited Major Pathological Response (MPR), and one patient showed PR. Currently, all four patients remain alive without evidence of tumor progression. Notably, the patient who achieved PCR has maintained a disease-free survival (DFS) exceeding 32 months post-surgery, while their event-free survival (EFS) has surpassed 36 months.

**Conclusions:**

Neoadjuvant immunotherapy in combination with chemotherapy has provided new promise for the treatment of locally advanced PSC with surgical potential. But these findings still need to be verified by further prospective researches.

## Introduction

PSC is a highly malignant and extremely heterogeneous tumor that falls under the category of NSCLC, exhibiting characteristics of both epithelial and mesenchymal tissue (1). It represents 1% of the overall incidence of NSCLC (2). According to the 2015 WHO classification of lung tumors, PSC can be categorized into five subtypes: pleomorphic carcinoma (PC), spindle cell carcinoma (SCC), giant cell carcinoma (GCC), carcinosarcoma (CS), and pulmonary blastoma (PB) (3). The prevalence is higher in elderly males, heavy smokers, as well as individuals exposed to dusty, chemical, or radiative environments for prolonged periods. Clinical presentations are primarily associated with tumor size, growth location, and local invasion. Tumors situated in the hilar region may lead to obstructive pneumonia or atelectasis with accompanying symptoms such as coughing, sputum production, hemoptysis, dyspnea and fever. Tumors located in the periphery of PSC patients typically present with either mild or no symptoms, but as the tumor progresses and infiltrates the pleura, it can lead to chest pain (4). The treatment principles for PSC align with those for other types of NSCLC; however, the therapeutic efficacy is suboptimal, and there is a high propensity for recurrence and metastasis. Among the 7,965 PSC patients statisticed by the US National Cancer Database from 2001 to 2011, the median survival for Stage I-II PSC was 16.9 months, for Stage III was 5.8 months, and for Stage IV was 5.4 months (5). Therefore, PSC urgently needs new treatment modalities to maximize its clinical benefits.

In recent years, significant progress has been made in the field of molecular targeted therapy for NSCLC, particularly in the research of molecular markers for PSC. These breakthroughs have provided a solid foundation for targeted treatment of PSC, offering new hope to patients. There have been documented cases of successful application of targeted drugs for PSC (6–8). Furthermore, immunotherapy has also advanced notably in the realm of lung cancer. Immune checkpoint inhibitors (ICI) such as PD-1 and PD-L1 blockers have demonstrated efficacy by activating the body’s immune system while impeding immune checkpoint functions, enabling T cells to combat tumor cells (9). ICI have received approval for multi-line treatment of NSCLC. The positive rate and expression level of PD-L1 in PSC are significantly higher than those observed in other forms of NSCLC, suggesting that PSC patients may derive benefits from immune checkpoint inhibitors (10). Reports indicated that the use of ICI holds promise for advanced PSC (11, 12). For resectable NSCLC, targeted therapy or immunotherapy has become an important strategy for perioperative treatment (13, 14). However, for resectable PSC, there have been no relevant studies. We present the treatment outcomes of four patients with locally advanced PSC who underwent neoadjuvant immunotherapy combined with platinum-based chemotherapy, all of whom successfully achieved R0 resection and demonstrated significant therapeutic benefits. Additionally, we report the latest advancements in targeted therapies and immunotherapies for PSC

## Case presentation

This study followed the guidelines of the Helsinki Declaration. The study protocol was reviewed and approved by the institutional review board and ethics committee of Shandong Cancer Hospital and Institute. Before treatment, all patients were provided with informed consent forms. The medical records were reviewed through the institution’s query system.

### Case 1

**Figure 1:**
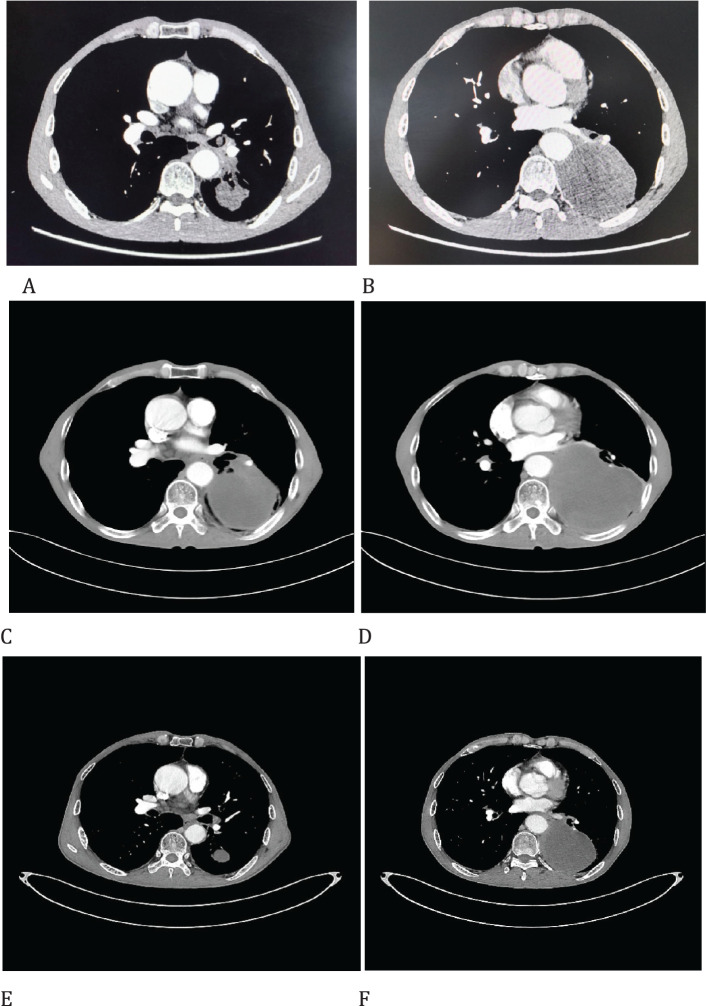
Imaging changes of patients before and after treatment. **(A, B)**, Baseline CT findings of the patient’s course (solid tumor, dmax = 90 mm * 70 mm, tumor obstructing the lower lobe bronchus). **(C, D)**, After targeted therapy (solid tumor, dmax = 120 mm * 43 mm, enlargement of lymph nodes in the hilum. **(E, F)**, After 2 cycles of immunotherapy plus chemotherapy (solid tumor, dmax = 46mm * 33mm, shrinking of hilar lymph nodes).

**Figure 2:**
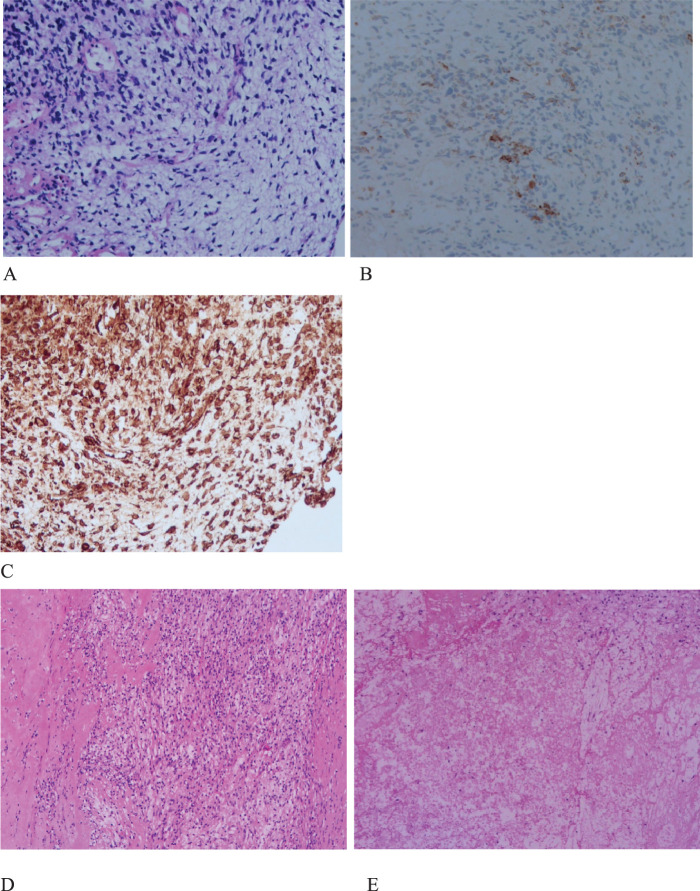
Histopathology and immunohistochemistry (IHC) of PSC. **(A)**, H&E stain, malignant spindle cell tumor. original magnification ×200. **(B)**, IHC CK (+), original magnification ×200. **(C)**, IHC Vimentin(+), original magnification ×200. **(D,E)**, extensive necrosis, fibrosis, inflammatory cell infiltration, and no evidence of malignant cells. original magnification ×100.

**Figure 3:**
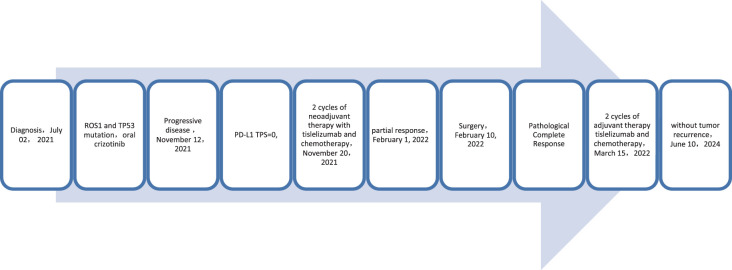
Timeline of treatment process of case 1.

A 62-year-old male patient presented to the Afliated Taian City Central Hospital of Qingdao University on July 02, 2021, with a persistent dry cough for 2 months. The enhanced chest CT scan revealed a 90mm*70mm tumor located in the lower lobe of the left lung, causing obstruction of the lower lobe bronchus (**Figures 1A, B**). The pathological findings from the transbronchial biopsy indicated a malignant spindle cell neoplasm. (**Figure 2A**) with immunohistochemistry (IHC) showed CK(+) and Vim(+) (**Figures 2B, C**). The initial diagnosis was PSC with a clinical staging of IIIA stage (cT4N0M0, 8th edition AJCC). The next generation sequencing (NGS) of tumor tissue samples to detect two mutations (ROS1 rearrangement with an abundance of 36.5% and TP53 mutation with an abundance of 10.86%). Therefore, treatment with crizotinib was initiated in July 2021. However, there was no improvement in the patient’s symptoms, and the chest pain progressively intensified, reaching a numerical pain score (NRS) of 10. The patient was admitted to our hospital in November 2021 for medical care. The enhanced chest CT scan shows that the tumor has grown larger compared to the previous examination, with a size of approximately 120mm*100mm and has invaded the pleura. Additionally, the lymph nodes in the hilum of the lungs are enlarged, suggesting a high likelihood of metastasis (**Figures 1C, D**). The Response Evaluation Criteria in Solid Tumors (RECIST) version 1.1 evaluation was PD. The PD-L1 (22C3) testing showed negative expression with a tumor proportion score (TPS) of 0. The patient received 2 cycles combination immunotherapy and platinum-based chemotherapy, specifically: tislelizumab 200mg d1 + paclitaxel for injection (albumin bound) 400mg d1 + Cisplatin 500mg d1 every three weeks. A follow-up chest CT showed PR (**Figures 1E, F**). Consequently, on February 10, 2022, left lower lobectomy and systemic lymph node dissection were carried out. The patient’s postoperative pathology report revealed extensive necrosis, fibrosis, and inflammatory cell infiltration with no residual cancer tissue detected (**Figures 2D, E**). These pathological changes were attributed to the treatment received. Regional lymph node status: 11 groups (0/7), 10 groups (0/3), 4L groups (0/1), 5 groups (0/6), 6 groups (0/2), 7 groups (0/2), 9 groups (0/2). The postoperative diagnosis was PSC (ypT0N0M0, PCR,8th edition AJCC). The patient received two cycles of adjuvant immunotherapy plus platinum-based chemotherapy. No immune-related adverse reactions were observed during treatment During postoperative follow-up, no recurrence was seen, with DFS exceeding 30 months and EFS exceeding 33 months (**Figure 3**).

### Case 2

**Figure 4:**
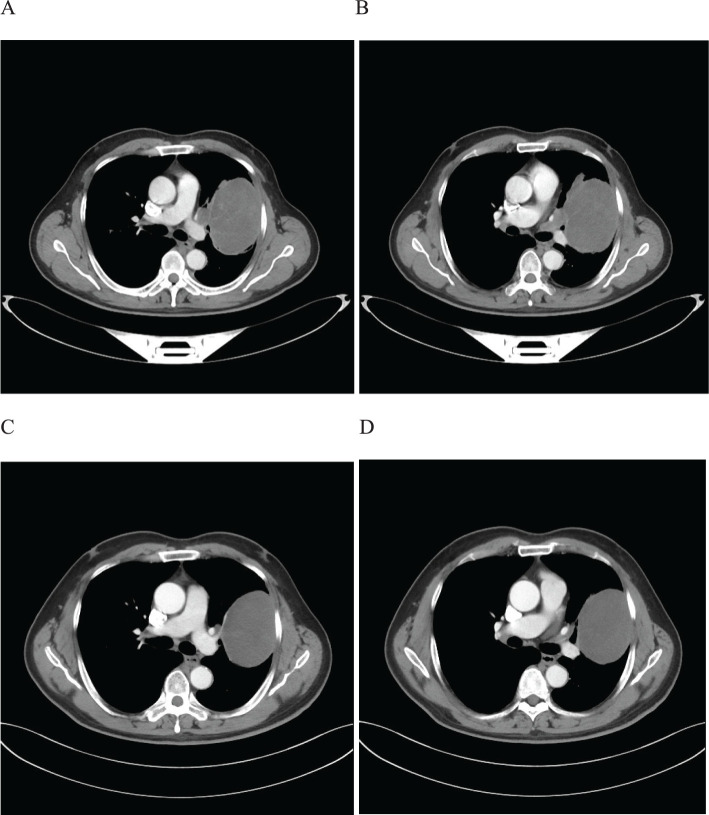
Imaging changes of patients before and after treatment. **(A, B)**, Baseline CT findings of the patient’s course (solid tumor, dmax = 90 mm * 60 mm, lymph nodes enlargement in the left hilar region, dmin= 17 mm). **(C, D)**, After 2 cycles of immunotherapy plus chemotherapy (solid tumor, dmax = 80mm * 60mm, shrinking of hilar lymph nodes).

**Figure 5:**
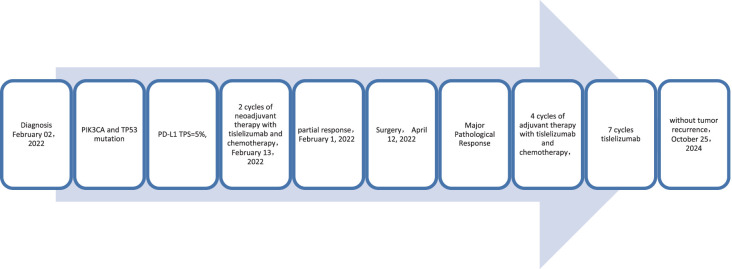
Timeline of treatment process of case 2.

A 59-year-old male patient presented to our hospital on February 06, 2022, with a persistent dry cough for 6 months. The enhanced chest CT scan reveals a 90mm*60mm tumor located in the upper lobe of the left lung with lymph node enlargement at the left hilar region, with a short diameter of 17 mm (**Figures 4A, B**). The pathological findings from the percutaneous lung biopsy indicated a malignant spindle cell neoplasm with IHC showed CKpan(+), Vim(+) and EMA(+). The initial diagnosis was PSC with a clinical staging of IIIA stage (cT4N1M0, 8th edition AJCC). The next generation sequencing (NGS) of tumor tissue samples to detect two mutations (PIK3CA mutation with an abundance of 7.1% and TP53 mutation with an abundance of 9.5%). The PD-L1 (22C3) testing showed negative expression with a tumor proportion score (TPS) of 5%. The patient received 2 cycles combination immunotherapy and platinum-based chemotherapy, specifically: tislelizumab 200mg d1 + paclitaxel for injection (albumin bound) 400mg d1 + Cisplatin 500mg d1 every three weeks, A follow-up chest CT with enhancement showed that the tumor volume and lymph nodes in the hilum had significantly decreased compared to before with (RECIST) version 1.1 evaluation showing PR (**Figures 4C, D**). Consequently, on April 12, 2022, left upper lobectomy and systemic lymph node dissection were carried out. The postoperative pathological results showed that about 10% of the tumor remained, which was classified as carcinosarcoma. The carcinoma component was of moderate differentiation squamous cell carcinoma, while the sarcoma component was rhabdomyosarcoma. Regional lymph node status: 11 groups (0/2), with one of the lymph nodes considered to have undergone changes following treatment. 10 groups (0/2), 4 groups (0/3), 5 groups (0/5), 6 groups (0/12), 7 groups (0/2), 9 groups (0/2). The postoperative diagnosis was PSC (ypTxN0M0, MPR,8th edition AJCC). The patient received 4 cycles of adjuvant immunotherapy plus platinum-based chemotherapy and 7 cycles of immunotherapy. No immune-related adverse reactions were observed during treatment. During postoperative follow-up, no recurrence was seen, with DFS exceeding 29 months and EFS exceeding 32 months (**Figure 5**).

### Case 3

**Figure 6:**
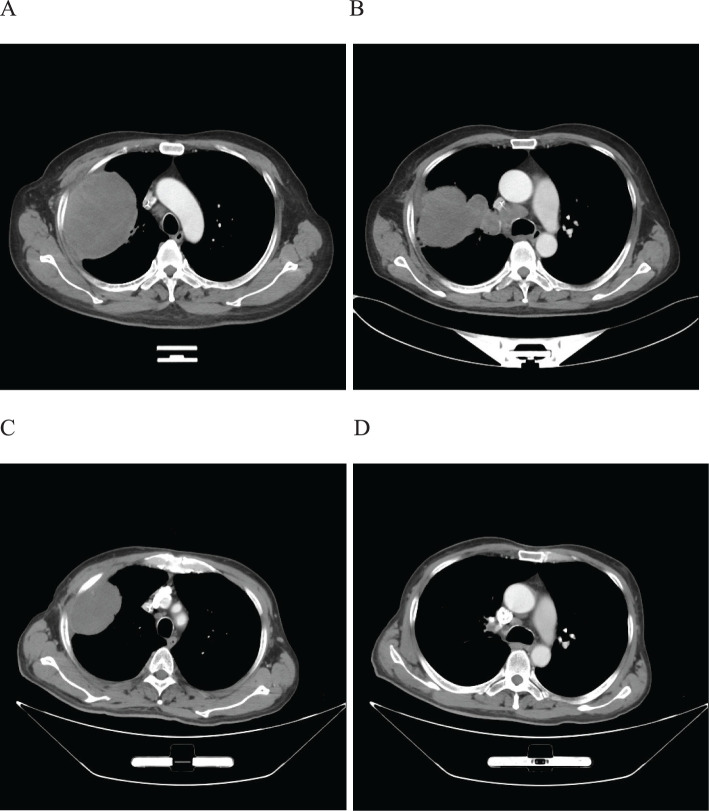
Imaging changes of patients before and after treatment. **(A, B)**, Baseline CT findings of the patient’s course (solid tumor, dmax = 80 mm * 60 mm, lymph nodes enlargement in the left hilar region and 4R group, dmin= 25 mm). **(C, D)**, After 4 cycles of immunotherapy plus chemotherapy (solid tumor, dmax = 80mm * 60mm, shrinking of lymph nodes).

**Figure 7:**
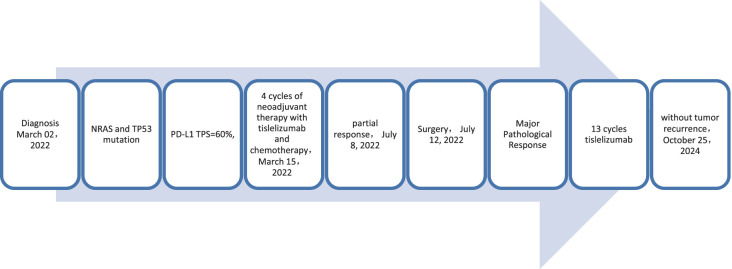
Timeline of treatment process of case 3.

A 56-year-old male patient presented to our hospital on March 30, 2022, with a persistent dry cough for 3 months. An enhanced chest CT scan demonstrated a tumor measuring 120mm x 90mm located in the upper lobe of the right lung, along with lymphadenopathy observed at the left hilar region and the 4R group where the largest node exhibited a short diameter of 25mm (**Figures 6A, B**). The pathological findings from the percutaneous lung biopsy indicated a malignant spindle cell neoplasm with IHC showed CK(+) and Vim(+).The initial diagnosis was PSC with a clinical staging of IIIB stage (cT4N2M0, 8th edition AJCC). The next generation sequencing (NGS) of tumor tissue samples to detect two mutations (NRAS mutation with an abundance of 20.2% and TP53 mutation with an abundance of 14.6%). The PD-L1 (22C3) testing showed negative expression with a tumor proportion score (TPS) of 60%. The patient received 4 cycles combination immunotherapy and platinum-based chemotherapy, specifically: tislelizumab 200mg d1 + paclitaxel for injection (albumin bound) 400mg d1 + Cisplatin 500mg d1 every three weeks, A follow-up chest CT with enhancement showed that the tumor volume and lymph nodes had significantly decreased compared to before with (RECIST) version 1.1 evaluation showing PR (**Figures 6C, D**). Consequently, on July 12, 2022, right upper lobectomy and systemic lymph node dissection were carried out. The postoperative pathological results showed that about 10% of the tumor remained, which was considered to be PSC. Regional lymph node status: 11 groups (0/4),10 groups (0/2), 2R group (0/1), 4R groups (0/2), 7 groups (0/2), 9 groups (0/2). The postoperative diagnosis was PSC (ypTxN0M0, MPR,8th edition AJCC). The patient received 13 cycles of immunotherapy. The patient did not show any recurrence during follow-up with DFS exceeding 27 months and EFS exceeding 31 months (**Figure 7**).

### Case 4

**Figure 8:**
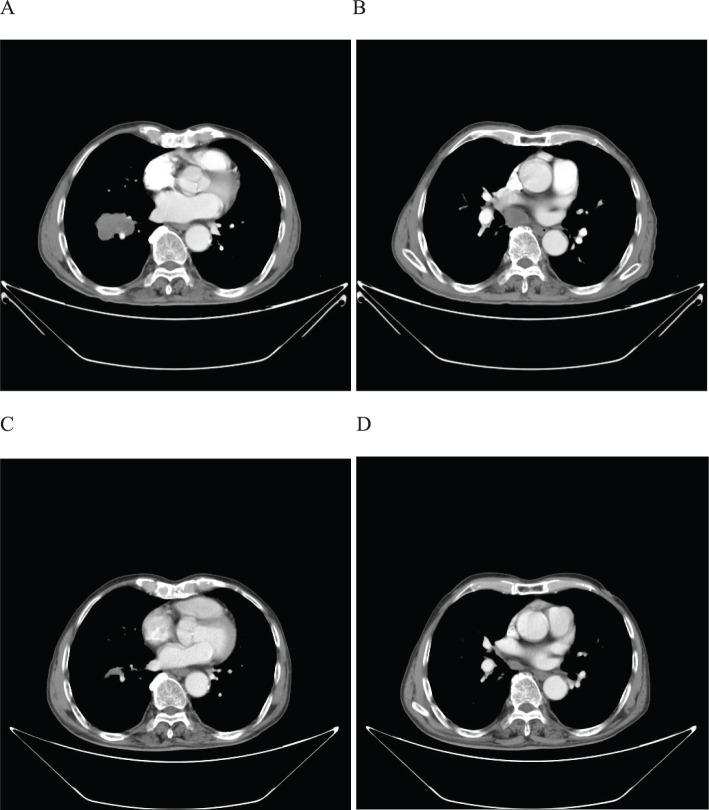
Imaging changes of patients before and after treatment. **(A, B)**, Baseline CT findings of the patient’s course (solid tumor, dmax = 48 mm * 37 mm, lymph nodes enlargement in the 7 group, dmin= 25 mm). **(C, D)**, After 2 cycles of immunotherapy plus chemotherapy (solid tumor, dmax = 24mm * 10mm, shrinking of lymph nodes in the 7 group).

**Figure 9:**
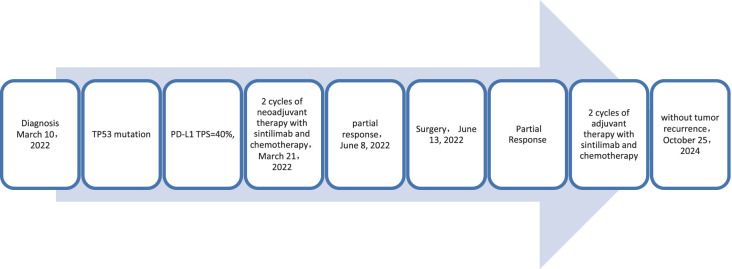
Timeline of treatment process of case 10.

A 65-year-old male patient presented to our hospital on March 10, 2022, with a persistent with a cough and hemoptysis for 1 month. The enhanced chest CT scan reveals a 48mm*37mm tumor located in the lower lobe of the right lung, causing obstruction of the lower lobe bronchus along with lymphadenopathy observed in the 7 group where the node exhibited a short diameter of 25mm (**Figures 8A, B**). The pathological findings from the percutaneous lung biopsy indicated a malignant spindle cell neoplasm with immunohistochemistry (IHC) showed CK pan(+), Vim(+) and CK7(+). The initial diagnosis was PSC with a clinical staging of IIIA stage (cT2N2M0, 8th edition AJCC). The next generation sequencing (NGS) of tumor tissue samples to detect TP53 mutation with an abundance of 47.6%. The PD-L1 (22C3) testing showed negative expression with a tumor proportion score (TPS) of 40%. The patient received 2 cycles combination immunotherapy and platinum-based chemotherapy, specifically: sintilimab 200 mg d1 + paclitaxel for injection (albumin bound) 400mg d1 + Cisplatin 500mg d1 every three weeks, A follow-up chest CT with enhancement showed that the tumor volume and lymph nodes in the 7 group had significantly decreased compared to before with (RECIST) version 1.1 evaluation showing PR (**Figures 8C, D**). Consequently, on Juny 13, 2022, right lower lobectomy and systemic lymph node dissection were carried out. The postoperative pathological results showed that about 30% of the tumor remained, which was considered to be PSC. Regional lymph node status: 11 groups (0/4), 10 groups (0/5), 4 groups (0/2), 2 groups (0/2), 7 groups (5/6), 9 groups (0/1). The postoperative diagnosis was PSC (ypTxN2M0, PR,8th edition AJCC). The patient received 2 cycles of adjuvant immunotherapy plus platinum-based chemotherapy. The patient did not show any recurrence during follow-up with DFS exceeding 30 months and EFS exceeding 33 months (**Figure 9**).

## Discussion

In comparison to NSCLC, PSC has a worse prognosis. A study utilizing surveillance, epidemiology, and end results databases tracked the prognosis of patients with PSC and found that this disease had significantly poorer overall and disease-specific survival compared to the population of conventional non-small cell lung cancer patients (15). Another extensive study using the National Cancer Database (NCDB) yielded similar unfavorable prognostic outcomes: it reported that the median survival period for PSC patients (6.4 months) was approximately half that of other non-small cell lung cancer patients, and this disparity in survival rates was statistically significant across all stages (5). Due to its rarity, research progress on PSC lags far behind other types of NSCLC. Given the bleak prognosis and lack of clear treatment guidelines, clinicians adopted a more pessimistic outlook on treatment and prognosis (16). Currently, evidence-based treatment strategies for PSC primarily consist of traditional methods such as surgery, radiotherapy, and chemotherapy.

A substantial number of PSC patients harbor targeted genomic mutations. Zhou et al. (12) conducted targeted gene analysis on 58 Chinese PSC patients, revealing gene mutations in 53 (91.4%) of them. Among the 175 patients included by Stephan-Falkenau et al., 61 (34.9%) had drug-accessible gene mutations (17). Reported gene mutations in PSC include TP53, KRAS, MET, EGFR, ALK, BRAF, PIK3CA, CDKN2A and PTEN. TP53 and KRAS exhibit a higher frequency of mutations in PSC, yet no targeted therapies have been reported for these two targets (12). There have been reports of PSC targeted therapies for MET, EGFR, ALK, and BRAF gene mutations. The MET14 exon skipping mutation is considered an adverse prognostic factor for advanced PSC patients. A multicenter clinical trial evaluating the efficacy of sevotinib in the treatment of NSCLC patients with MET14 exon skipping mutation enrolled 25 PSC patients, demonstrating an objective response rate of up to 40% and a median response duration of 17.9 months, a median progression-free survival (PFS) of 5.5 months, and mostly grade 1-2 treatment-related adverse reactions (6). This finding suggests that sevotinib demonstrates favorable efficacy and safety in PSC patients with MET14 exon skipping mutation. Currently, targeted therapies for EGFR, ALK, and BRAF targets are limited to case reports and have shown good clinical effects (7, 18–20). The patient in case 1 was found to have ROS1 rearrangement and TP53 mutation. ROS1 rearrangement in PSC has been rarely reported. C. Xu et al. included a total of 35 PSC patients, among whom three were detected to have ROS1 rearrangements by reverse transcription polymerase chain reaction. The incidence of ROS1 rearrangement in Chinese PSC patients was found to be higher than that in other subtypes of NSCLC. Crizotinib may represent an effective treatment option for ROS1 rearrangement-positive PSC (21). In case 1, the patient exhibited tumor progression during treatment with crizotinib. Previous studies have demonstrated that co-mutations of TP53 with ROS1 or EGFR significantly diminish the efficacy of targeted therapies (22). We believe that the patient developed primary resistance to crizotinib due to the coexistence of TP53 mutation. Given that TP53 mutation (74%) are common in PSC (12), the efficacy of targeted therapy may be lower compared to other types of NSCLC. Further research is warranted to identify the specific population that can benefit from targeted therapy in the future.

Biomarkers play an important role in predicting the efficacy of immunotherapy. We assessed the potential value of immunotherapy in PSC by analyzing the expression of biomarkers in PSC. PD-L1 expression currently stands as the most well-established biomarker for predicting the response and clinical efficacy of ICI in NSCLC (23). Similar to other NSCLC, high expression of PD-L1 is associated with better treatment outcomes (24). Babacan et al. (25)] analyzed the efficacy of ICI in 66 PSC patients with PD-L1 expression, with an objective response rate of 70.2% for 49 patients with PD-L1≥50%, a 50% objective response rate for 10 patients with PD-L1 of 1% to 49%, and a 28.6% objective response rate for 7 patients with PD-L1<1%. In a study of 22 patients with PSC exhibiting PD-L1 expression of ≥50%, pembrolizumab monotherapy demonstrated an objective response rate and disease control rate of 68.2% and 81.8%, respectively, indicating the potential efficacy of immune monotherapy for high PD-L1 expressing PSC (26). To our surprise, the positive rate and high expression rate of PD-L1 in the PSC were significantly higher than other NSCLC. Lee et al. (27) studied 125 PSC patients for PD-L1 expression, with 89.6% (112/125) testing positive and 80.0% (100/125) showing high levels of expression. Ma et al. (28) analyzed 32 PSC patients, observing that 59.4% (19/32) expressed PD-L1 positively, while 34.4% (11/32) exhibited greater than 50% expression. Due to the tendency of high expression of PD-L1, we believe that ICI has a broad application prospect in PSC patients. However, in clinical practice, some patients with high PD-L1 expression do not respond to immunotherapy, and in fact, some may even experience hyperprogression (HPD). Fricke J et al. (29) reported a case of advanced NSCLC patient with 100% PD-L1 expression who experienced HPD after two cycles of pembrolizumab treatment, which was controlled by chemotherapy and allowed for long-term stability. Oguri T et al. (30) reported a case of advanced PSC patient with high PD-L1 expression (90%) who died of HPD 13 days after receiving atezolizumab. PD-L1 expression alone may not reliably predict the efficacy of immunotherapy.

Gene mutations also frequently serve as crucial biomarkers for the effectiveness of immunotherapy. Fricke J et al. thought HPD of PSC may be associated with STK11 mutation (29). A recent study has shown that there is an association between STK11 and HPD in NSCLC patients, with (3/16) of the patients with HPD having STK11 mutations, while (0/28) of the patients without HPD were not found to have STK11 mutations (31). STK11 mutation have been linked to a diminished response to immunotherapy and poor prognosis (32). In addition to STK11 mutation, ICIs have limited efficacy for NSCLC with EGFR, HER2, ALK, ROS1, MET or RET mutations (33, 34). ICIs show good efficacy for NSCLC with TP53 gene mutations in NSCLC. Wu et al.’s study showed that patients with TP53 mutations had higher tumor mutation burden (TMB) and PD-L1 expression levels than those with wild-type TP53, and were associated with better median PFS (35). High prevalence of TP53 gene mutations in PSC suggest that immunotherapy may confer substantial clinical benefits to patients. Single KRAS gene mutation does not exhibit a significant correlation with the efficacy of immunotherapy except for PD-L1 is highly expressed (36, 37). However, co-mutations of KRAS and TP53 have been shown to enhance the efficacy of immunotherapy in NSCLC. A study of 696 patients with NSCLC who received pembrolizumab as first-line treatment revealed that the co- mutation of TP53 and KRAS G12C was associated with the longest PFS and OS, surpassing outcomes associated with single mutation or wild-type status (38). Terra SB et al. included 33 PSC patients in their study and found that 6 of them had a TP53/KRAS co-mutation (18%) (39).

TMB is an emerging biomarker for immunotherapy, in addition to PD-L1 expression. The correlation between PD-L1 expression and TMB is not significant in lung cancer, and assessing TMB can expand the patient population that may benefit from immunotherapy. In the CheckMate-026 study, high TMB ((10 variants/Mb) NSCLC patients receiving Nivolumab as first-line treatment demonstrated an ORR of 47%, compared to only 28% for those receiving chemotherapy, with a median PFS of 9.7 months and 5.8 months respectively. Conversely, middle and low TMB patients showed significantly worse median PFS when receiving immunotherapy compared to chemotherapy (40). Schrock et al. observed that more than 60% of patients with PSC exhibit high TMB (20). The median TMB in PSC is 8.1 mutations/Mb (12), with 37.9-87.5% of Chinese PSC patients having high TMB (12, 41). This suggests that PSC may benefit from ICIs.

Currently, the effect of immunotherapy in advanced PSC is very significant. Domblides et al. (42) observed that among 37 PSC patients who received nivolumab as a second-line or beyond second-line treatment after platinum-based chemotherapy, irrespective of PD-L1 status, there was an objective response rate of 40.5% and a disease control rate of 64.8%, with median OS of 12.7 months. Babacan NA et al. (25) reported that among the 90 PSC patients who received immune checkpoint inhibitor treatment, the rate of complete remission (CR) and partial remission (PR) was 54.5%, and the rate of disease stability (SD) was 15.9%, while the rate of disease progression (PD) was 29.6%. The median progression-free survival (PFS) was 7.0 months. Immunotherapy mainly consists of two modes: single-agent immunotherapy and combined immunotherapy alongside chemotherapy. Nevertheless, multiple clinical trials like KEYNOTE-024 have demonstrated that single-agent immunotherapy confers clinical benefits exclusively on patients exhibiting high PD-L1 expression (43–45). Consequently, it is apparent that standalone immunotherapeutic interventions are inadequate in addressing clinical needs. The combination of immunotherapy and chemotherapy has demonstrated superior efficacy compared to the use of immunotherapy alone, conferring benefits to patients irrespective of their PD-L1 expression level (46–49). Studies like Checkmate-816 and Keynote-671 have shown that immunotherapy combined with chemotherapy has a significant effect in resectable NSCLC, with high PCR rates and longer EFS (13, 14). In the Rationale 315 study, the combination of tislelizumab with chemotherapy achieved a PCR rate of up to 40.7% and an MPR rate of up to 56.2%, while significantly reducing the risk of disease recurrence and progression (HR for EFS: 0.56, 95% CI: 0.40-0.79). Additionally, it showed good tumor shrinkage, with an objective response rate of 71.2%, thereby reducing the difficulty of surgery (50). Given its significant therapeutic effect, we applied this treatment pattern to patients with locally advanced PSC. After the progression of the tumor on crizotinib therapy in case 1, we decided to initiate the treatment regimen of tislelizumab combined with chemotherapy. After 2 cycles of this combination therapy, a significant reduction in chest pain was observed in this patient, leading to an enhancement in their quality of life. Simultaneously, tumor downstaging occurred, reducing surgical complexity. The patient’s PCR test results indicated a high likelihood of survival. Currently, The EFS has surpassed 36 months, while the DFS after surgery has exceeded 32 months. Encouraged by the remarkable success achieved with neoadjuvant immunotherapy combined with chemotherapy in this case, we subsequently administered this regimen to three patients. Among them, 2 patients achieved complete pathologic response (MPR) and 1 patient achieved partial response (PR), with no evidence of recurrence detected during postoperative follow-up; Neoadjuvant immunotherapy combined with chemotherapy holds promise as a strategy for treating resectable PSC. However, due to its low incidence and limited surgical opportunities further prospective multicenter studies are warranted to validate ICI efficacy.

## Conclusion

Given the high mutation rate of TP53 in PSC, the effectiveness of targeted therapy may be limited; thus, further research is essential to identify specific patient populations that could benefit from such interventions. Biomarkers including PDL1, TMB, and TP53 are significantly elevated in PSC, indicating substantial potential for immune checkpoint inhibitors in clinical applications. A combination of neoadjuvant immunotherapy and chemotherapy may be a promising strategy for treating potentially resectable PSC. However, its effectiveness and safety still need to be validated in the future.

## Data Availability

The original contributions presented in the study are included in the article/**Ssupplementary Material**. Further inquiries can be directed to the corresponding author.
